# Determinants of extended inpatient care in odontogenic cervicofacial infections: a retrospective observational study

**DOI:** 10.1186/s12903-026-10392-5

**Published:** 2026-11-03

**Authors:** Anna Aydin, Lawik Revend, Doha Revend, Thomas Beikler, Manfred Giese, Oliver Schuck, Johannes Schunk, Florian Dudde

**Affiliations:** 1 Department of Oral and Maxillofacial Surgery, Army Hospital Hamburg, Lesserstraße 180, Hamburg, 22049 Germany; 2 Department of Plastic Surgery, Army Hospital Berlin, Berlin, Germany; 3 Department of Otolaryngology – Head and Neck Surgery, Army Hospital Berlin, Berlin, Germany; 4 Department of Periodontics, Preventive and Restorative Dentistry, University Medical Center Hamburg-Eppendorf, Hamburg, Germany

**Keywords:** Odontogenic infection, Risk factor, Extended inpatient care, Length of stay

## Abstract

**Background:**

This study aimed to identify clinical and demographic factors associated with prolonged hospitalisation in patients treated for severe odontogenic cervicofacial infections (CFI).

**Methods:**

This retrospective study analysed 152 patients who were treated in an inpatient setting for CFI at the Department of Oral and Maxillofacial Surgery, Army Hospital Hamburg, between January 2022 and December 2023. Patients were stratified based on length of hospital stay (LOS) into two groups: <7 days and ≥ 7 days. Demographic, clinical, laboratory, treatment-related and timing data were compared to determine their association with prolonged LOS.

**Results:**

Patients with prolonged hospitalisation (≥ 7 days; *n* = 79) were older and more frequently had recorded pre-existing disease, multi-space infection, submandibular or floor-of-mouth involvement, dyspnoea, elevated inflammatory markers and delayed presentation in unadjusted analyses. In multivariable logistic regression, recorded pre-existing disease (aOR 6.04, 95% CI 1.10–33.18; *p* = 0.038) and longer time from symptom onset to hospital presentation (aOR 2.86 per day, 95% CI 2.01–4.07; *p* < 0.001) remained independently associated with prolonged hospitalisation.

**Conclusions:**

Although several demographic, anatomical and clinical factors were associated with prolonged hospitalisation in unadjusted analyses, recorded pre-existing disease and delayed hospital presentation remained independently associated with prolonged LOS after multivariable adjustment. These findings highlight the potential importance of comorbidity and timely presentation in identifying patients at risk of extended inpatient care, but prospective multicentre validation is warranted.

## Introduction

Odontogenic infections represent a frequent and clinically significant group of conditions encountered in oral and maxillofacial surgery [1]. They originate from dental or periodontal sources and can spread into adjacent cervicofacial spaces [2, 3]. While many remain localised and can be managed in an outpatient setting, severe infections may necessitate hospitalisation, surgical drainage, intravenous antimicrobial therapy and, occasionally, airway or intensive-care support [3–8]. Airway compromise, deep-neck extension, mediastinitis, sepsis and death remain recognised complications [2, 5, 9]. Contemporary German data confirm a continuing burden of severe maxillofacial space infections [10, 11].

Prolonged hospitalisation is not only a marker of clinical severity but also contributes to healthcare costs, patient morbidity and resource utilisation. Previous studies have associated longer admission with older age, comorbidity, diabetes, multi-space involvement and inflammatory markers [12–17]. Recent cohorts have further emphasised the roles of time to surgery [18], multi-space disease [19–21], nutritional and inflammatory status [20], and age or cardiometabolic comorbidity [19, 21, 22]. Recent adjusted analyses have also identified infection extent, disease severity and systemic comorbidity as independent determinants of prolonged hospitalisation in comparable inpatient cohorts [23, 24]. However, definitions of prolonged stay, inclusion criteria and analytical approaches vary, supporting renewed evaluation in a contemporary odontogenic-only cohort.

Against this background, the present study aimed to investigate demographic, clinical, anatomical, laboratory, timing and treatment-related factors associated with prolonged hospital stay in patients treated for odontogenic CFI. The findings are intended to support early risk stratification, clinical decision-making and resource planning.

## Materials and methods

### Study design and participants

This retrospective observational study included 152 patients who were treated for odontogenic cervicofacial infections (CFI) in the Department of Oral and Maxillofacial Surgery between 1 January 2022 and 31 December 2023. Inclusion criteria comprised patients aged ≥ 18 years who required inpatient treatment due to odontogenic infection. Patients with non-odontogenic infections or incomplete records were excluded. Reporting follows the STROBE recommendations for observational studies [25].

### Clinical management

All patients underwent standardised diagnostic work-up, including clinical examination, laboratory testing (C-reactive protein [CRP] and white blood cell count [WBC]), and imaging (panoramic radiographs and/or contrast-enhanced computed tomography [CT] scans). Treatment consisted of intravenous antibiotics, surgical incision and drainage (intraoral and/or extraoral), and dental extraction as indicated. Antibiotic agent, microbiology-guided adjustment and treatment duration were not evaluated as study variables; a uniform regimen was therefore not assumed. Airway management, including tracheostomy and intensive care unit (ICU) admission, was performed when clinically required. The timing and type of intervention were documented for each patient.

### Data collection

Data were extracted from electronic medical records and included demographic variables (age, sex), separately coded comorbidity variables (recorded pre-existing disease, diabetes and cardiac disease), smoking status, anatomical location and type of infection, presenting symptoms (fever, dyspnoea, trismus, dysphagia), laboratory values (CRP, WBC), type of surgical intervention, timing of care (symptom onset to hospital presentation and operation), and treatment course (ICU admission, tracheostomy, dental management). The comorbidity fields could overlap and were not treated as a hierarchical composite. Dyspnoea, dysphagia and trismus were extracted as documented at admission; uniform quantitative thresholds were not applied in the retrospective analysis.

### Length of stay and grouping

Patients were stratified into two groups based on length of hospital stay (LOS): short stay, < 7 days, versus prolonged stay, ≥ 7 days. The 7-day cut-off was used in the original analysis and separated the cohort into two comparably sized groups.

### Statistical analysis

Statistical analysis was conducted using SPSS (version 28.0, IBM Corp., Armonk, NY, USA). Continuous variables were expressed as mean ± standard deviation and compared using the independent-samples t-test. Categorical variables were compared using the chi-square test or Fisher’s exact test where appropriate. A p value < 0.05 was considered statistically significant. To visualise differences between groups, boxplots were generated as graphical illustrations. A multivariable binary logistic regression analysis was performed to identify factors independently associated with prolonged hospitalisation (LOS ≥ 7 days). Eight clinically relevant variables representing demographic characteristics, comorbidity, anatomical extent, clinical severity, inflammatory burden and timing of presentation were entered simultaneously into the model: age, recorded pre-existing disease, multi-space infection, submandibular space abscess, floor-of-mouth abscess, dyspnoea, CRP and time from symptom onset to hospital presentation. Adjusted odds ratios (aORs) with 95% confidence intervals (CIs) were reported. Model fit was assessed using the Hosmer–Lemeshow goodness-of-fit test and Nagelkerke’s R². Multicollinearity among the included predictors was assessed using variance inflation factors (VIFs), with no evidence of problematic multicollinearity (all VIFs < 2.1).

## Results

### Baseline characteristics

A total of 152 patients were included in the analysis. The overall mean age was 54.9 ± 19.5 years (Table 1). Female patients represented a slight majority (53.3%) (Table 1). When comparing the two groups based on length of hospital stay (LOS), patients with a prolonged stay of ≥ 7 days (*n* = 79) were significantly older than those with a shorter stay of < 7 days (*n* = 73), with mean ages of 58.6 ± 20.7 and 50.9 ± 17.4 years, respectively (*p* = 0.015) (Table 1). Age > 60 years was likewise more frequent in the prolonged-LOS group (67.1% versus 34.2%; *p* < 0.001) (Table 2).

**Table 1:** Baseline characteristics

Variable	Total (*n* = 152)	LOS ≥ 7 days (*n* = 79)	LOS < 7 days (*n* = 73)	*p* value
Age (years)	54.91 (± 19.52)	58.59 (± 20.71)	50.92 (± 17.42)	**0.015**
Sex				0.345
Male	71 (46.7)	34 (43.0)	37 (50.7)	
Female	81 (53.3)	45 (57.0)	36 (49.3)	

Data are presented as mean (SD) or *n* (%). Significant values are presented in bold

**Table 2:** Clinical and anatomical characteristics of odontogenic CFI

Variable	Total (*n* = 152)	LOS ≥ 7 days (*n* = 79)	LOS < 7 days (*n* = 73)	*p* value
Canine space abscess (fossa canina)	6 (3.9)	4 (5.1)	2 (2.7)	0.683
Buccal/maxillary space abscess	20 (13.2)	10 (12.7)	10 (13.7)	0.850
Retromaxillary space abscess	8 (5.3)	4 (5.1)	4 (5.5)	1.000
Paramandibular space abscess	35 (23.0)	13 (16.5)	22 (30.1)	**0.045**
Perimandibular space abscess	44 (28.9)	27 (34.2)	17 (23.3)	0.139
Submandibular space abscess	40 (26.3)	28 (35.4)	12 (16.4)	**0.008**
Floor-of-mouth abscess	28 (18.4)	23 (29.1)	5 (6.8)	**< 0.001**
Pterygomandibular space abscess	8 (5.3)	4 (5.1)	4 (5.5)	1.000
Symptoms				
Fever	112 (73.7)	69 (87.3)	43 (58.9)	**< 0.001**
Dyspnoea	83 (54.6)	50 (63.3)	33 (45.2)	**0.025**
Dysphagia	107 (70.4)	55 (69.6)	52 (71.2)	0.828
Pain	152 (100.0)	79 (100.0)	73 (100.0)	/
Swelling	152 (100.0)	79 (100.0)	73 (100.0)	/
Trismus	94 (61.8)	49 (62.0)	45 (61.6)	0.961
Single-space infection	120 (78.9)	53 (67.1)	67 (91.8)	**< 0.001**
Multi-space infection	32 (21.1)	26 (32.9)	6 (8.2)	**< 0.001**
Age > 60 years	78 (51.3)	53 (67.1)	25 (34.2)	**< 0.001**
Smoker	74 (48.7)	36 (45.6)	38 (52.1)	0.424
Recorded pre-existing disease	63 (41.4)	44 (55.7)	19 (26.0)	**< 0.001**
Diabetes	60 (39.5)	37 (46.8)	23 (31.5)	0.053
Cardiac disease	69 (45.4)	45 (57.0)	24 (32.9)	**0.003**

Data are presented as *n* (%). Significant values are presented in bold. Anatomical labels reproduce the categories used in the source database; paramandibular, perimandibular and retromaxillary spaces were retained as separate categories

There was no significant difference in sex distribution between the two groups (*p* = 0.345) (Table 1). However, recorded pre-existing disease was more frequent in the prolonged group (55.7% versus 26.0%; *p* < 0.001) (Table 2). Cardiac disease was also significantly more common in the prolonged group (57.0% versus 32.9%; *p* = 0.003), while diabetes showed a non-significant trend towards increased prevalence (46.8% versus 31.5%; *p* = 0.053) (Table 2).

### Infection characteristics and symptoms

Regarding the anatomical distribution of infections, submandibular abscesses were significantly more frequent in the prolonged-LOS group (35.4%) than in the short-stay group (16.4%) (*p* = 0.008) (Table 2). Likewise, floor-of-mouth abscesses were associated with longer hospital stays (29.1% versus 6.8%; *p* < 0.001) (Table 2). Multi-space infections were present in 32.9% of patients with prolonged LOS, compared with 8.2% in those with shorter hospitalisation (*p* < 0.001) (Table 2). Conversely, single-space infections were more often observed in short-stay patients (91.8% versus 67.1%; *p* < 0.001). Fever was more frequent in patients with prolonged hospitalisation (87.3% versus 58.9%; *p* < 0.001), as was dyspnoea (63.3% versus 45.2%; *p* = 0.025). Dysphagia, trismus, pain and swelling did not differ significantly between the groups (Table 2).

### Laboratory and therapeutic factors

In terms of laboratory parameters, patients in the prolonged-LOS group showed significantly higher inflammatory markers: the mean CRP level was 87.4 ± 70.7 mg/L versus 65.5 ± 48.7 mg/L in the short-stay group (*p* = 0.029), and the WBC was 15.5 ± 5.2 × 10⁹/L versus 13.5 ± 3.7 × 10⁹/L (*p* = 0.007) (Table 3).

**Table 3:** Laboratory and treatment-course factors associated with prolonged hospitalisation

Variable	Total (*n* = 152)	LOS ≥ 7 days (*n* = 79)	LOS < 7 days (*n* = 73)	*p* value
CRP (mg/L)	76.88 (± 61.94)	87.41 (± 70.71)	65.49 (± 48.74)	**0.029**
WBC (×10⁹/L)	14.52 (± 4.64)	15.49 (± 5.17)	13.48 (± 3.74)	**0.007**
Intraoral incision and drainage	89 (58.6)	54 (68.4)	35 (47.9)	**0.011**
Extraoral incision and drainage	63 (41.4)	25 (31.6)	38 (52.1)	
Tracheostomy	18 (11.8)	16 (20.3)	2 (2.7)	**< 0.001**
ICU	22 (14.5)	20 (25.3)	2 (2.7)	**< 0.001**
Immediate tooth extraction	28 (18.4)	5 (6.3)	23 (31.5)	**< 0.001**
Delayed tooth extraction during follow-up	78 (51.3)	54 (68.4)	24 (32.9)	**< 0.001**
Conservative dental treatment	45 (29.6)	20 (25.3)	25 (34.2)	0.228

Data are presented as mean (SD) or *n* (%). Significant values are presented in bold. Drainage route is one mutually exclusive comparison​

*WBC* White blood cell count, *CRP* C-reactive protein, *ICU*  Intensive care unit

**Fig. 1:**
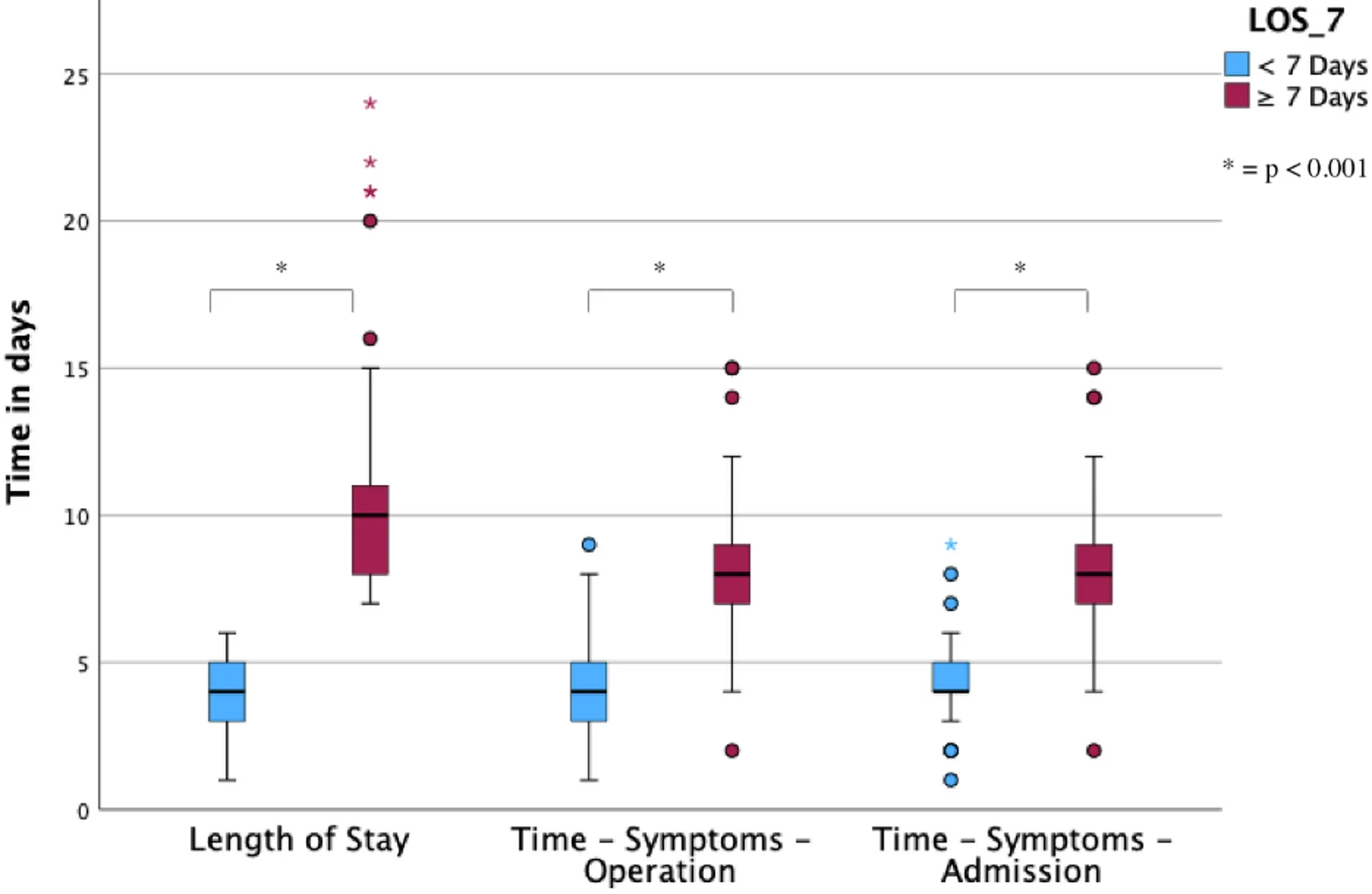
Timing intervals and length of stay by hospital-stay group. Boxplots show LOS, time from symptom onset to hospital presentation and time from symptom onset to operation for LOS < 7 days and LOS ≥ 7 days. Centre lines indicate medians, boxes the interquartile range, whiskers values within 1.5 interquartile ranges, circles outliers and asterisks extreme values. Brackets denote between-group comparisons; *p* < 0.001

Treatment modalities also differed between groups. Drainage route differed between the LOS groups (*p* = 0.011): intraoral incision and drainage was more frequent in the prolonged group (68.4% versus 47.9%), whereas extraoral drainage was more frequent in the short-stay group (52.1% versus 31.6%) (Table 3). ICU admission (25.3% versus 2.7%; *p* < 0.001) and tracheostomy (20.3% versus 2.7%; *p* < 0.001) were substantially more common among patients with extended stays (Table 3). Immediate dental extractions were significantly more frequent in the short-stay group (31.5% versus 6.3%; *p* < 0.001), while postponed extractions during follow-up were more frequent in patients with longer hospitalisation (68.4% versus 32.9%; *p* < 0.001) (Table 3). Conservative dental treatment did not differ significantly between groups (*p* = 0.228) (Table 3). Patients with prolonged hospital stays experienced longer intervals between symptom onset and hospital presentation (8.00 ± 2.41 versus 4.26 ± 1.33 days; *p* < 0.001) and between symptom onset and operation (8.01 ± 2.44 versus 4.27 ± 1.32 days; *p* < 0.001) (Fig. 1; Table 4). The near-identical day-level intervals indicate that a separate in-hospital delay cannot be resolved from these summary variables. Mean LOS was 10.4 ± 3.7 days in the prolonged group versus 4.2 ± 1.3 days in the short-stay group (*p* < 0.001) (Fig. 1; Table 4).

**Table 4:** Timing of clinical events in relation to length of hospital stay

Variable	Total (*n* = 152)	LOS ≥ 7 days (*n* = 79)	LOS < 7 days (*n* = 73)	*p* value
Symptom onset to hospital presentation, days	6.20 (± 2.71)	8.00 (± 2.41)	4.26 (± 1.33)	**< 0.001**
Symptom onset to operation, days	6.21 (± 2.72)	8.01 (± 2.44)	4.27 (± 1.32)	**< 0.001**
Length of stay, days	7.39 (± 4.22)	10.39 (± 3.74)	4.15 (± 1.30)	**< 0.001**

Data are presented as mean (SD) in days. Significant values are presented in bold

A multivariable binary logistic regression analysis was performed to identify factors independently associated with prolonged hospitalisation (LOS ≥ 7 days). The overall model was statistically significant (χ²(8) = 105.56, *p* < 0.001), with a Nagelkerke R² of 0.691. The Hosmer–Lemeshow test indicated no evidence of poor model fit (χ²(8) = 6.92, *p* = 0.546). After simultaneous adjustment for age, recorded pre-existing disease, multi-space infection, submandibular and floor-of-mouth involvement, dyspnoea, CRP and time from symptom onset to hospital presentation, two variables remained independently associated with prolonged hospitalisation (Table 5). Recorded pre-existing disease was associated with higher odds of prolonged hospitalisation (aOR 6.04, 95% CI 1.10–33.18; *p* = 0.038), although the wide confidence interval indicated substantial uncertainty in the magnitude of this association. Each additional day between symptom onset and hospital presentation was associated with higher odds of prolonged hospitalisation (aOR 2.86, 95% CI 2.01–4.07; *p* < 0.001). Age, multi-space infection, submandibular and floor-of-mouth involvement, dyspnoea and CRP were not independently associated with prolonged hospitalisation after adjustment (all *p* > 0.05).

**Table 5:** Multivariable logistic regression analysis of factors associated with prolonged hospitalisation (LOS ≥ 7 days)

Variable	Adjusted OR	95% CI	*p* - Value
Age (year)	0.98	0.94–1.03	0.451
Recorded pre-existing disease	6.04	1.10–33.18	**0.038**
Multi-space infection	3.19	0.33–31.32	0.319
Submandibular space abscess	1.20	0.26–5.57	0.817
Floor-of-mouth abscess	0.94	0.08–10.92	0.959
Dyspnoea	1.22	0.38–3.93	0.743
CRP (mg/L)	1.00	0.99–1.02	0.128
Symptom onset to hospital presentation, days	2.86	2.01–4.07	**< 0.001**

Adjusted odds ratios were obtained from a multivariable binary logistic regression model including all variables shown. The dependent variable was prolonged hospitalisation (LOS ≥ 7 days). Significant values are presented in bold

*OR* Odds ratio, *CI* Confidence interval, *CRP* C-reactive protein

## Discussion

This study investigated clinical and demographic factors associated with prolonged hospitalisation in patients with odontogenic cervicofacial infections. Given the resource use associated with severe infections in emergency, surgical and intensive-care settings, the aim was to identify variables that may support early risk stratification and management planning. In unadjusted analyses, older age, recorded comorbidity, anatomical spread, dyspnoea, inflammatory markers and delayed presentation were associated with prolonged LOS. However, after simultaneous multivariable adjustment, only recorded pre-existing disease and time from symptom onset to hospital presentation remained independently associated with prolonged hospitalisation. In particular, each additional day between symptom onset and hospital presentation was associated with substantially higher odds of prolonged hospitalisation. These findings suggest that several characteristics associated with prolonged LOS in unadjusted comparisons may represent interrelated markers of disease severity rather than independent determinants of extended inpatient care. Dyspnoea was significantly associated with prolonged hospitalisation in the unadjusted analysis, whereas dysphagia was similarly frequent in both groups and was not associated with LOS. Dyspnoea may reflect advanced infection involving airway-relevant spaces and therefore remains a clinically important warning sign [6, 26, 27]. Its recognition should prompt timely imaging, surgical assessment and consideration of airway monitoring [9, 26–28]. However, dyspnoea did not remain independently associated with prolonged LOS after adjustment for the other variables included in the multivariable model. This finding does not diminish its clinical relevance for airway assessment but suggests that its unadjusted association with LOS may partly reflect overall disease severity.

The anatomical extent of infection also appeared relevant in the unadjusted analyses. Infections confined to superficial spaces, such as the buccal or canine space, often follow a less complex course [28–30], whereas submandibular, floor-of-mouth and multi-space infections may threaten the airway and require broader surgical exposure or intensive monitoring [9, 26, 27]. In the prospective outcomes study by Flynn et al., the number of infected spaces predicted LOS [31]. In the present multivariable model, however, multi-space infection, submandibular involvement and floor-of-mouth involvement were no longer independently associated with prolonged LOS. Their significant unadjusted associations may therefore partly reflect the overall severity and complexity of infection rather than independent effects on hospital stay.The association with delayed presentation was particularly pronounced. Patients with prolonged LOS had substantially longer symptom-to-presentation intervals, and this association persisted after adjustment for age, recorded pre-existing disease, anatomical extent, dyspnoea and CRP. Each additional day between symptom onset and hospital presentation was associated with higher odds of prolonged hospitalisation (aOR 2.86, 95% CI 2.01–4.07). Delayed presentation may permit further progression of odontogenic infection and thereby increase the complexity of subsequent inpatient management. Nevertheless, given the retrospective observational design, this association should not be interpreted as evidence of causality. The timing variables were analysed continuously; no categorical > 5-day threshold was evaluated. Moreover, the symptom-to-presentation and symptom-to-operation intervals were nearly identical at day-level resolution and therefore do not provide a separate estimate of in-hospital surgical delay, although such delay may influence LOS in other cohorts [18].

Age was significantly associated with prolonged LOS in the unadjusted analyses, both as a continuous variable and when patients aged > 60 years were considered. Recorded pre-existing disease and cardiac disease were also more frequent in the prolonged-LOS group, whereas sex and smoking status were not associated. These findings are consistent with previous studies linking longer hospitalisation to age and systemic comorbidity [12, 16, 19, 21, 22]. After multivariable adjustment, age was no longer independently associated with prolonged hospitalisation, whereas recorded pre-existing disease remained significant (aOR 6.04, 95% CI 1.10–33.18). The wide confidence interval indicates considerable uncertainty regarding the magnitude of this association and warrants cautious interpretation. Nevertheless, the finding supports the relevance of systemic comorbidity when assessing the expected complexity of the inpatient course.

The association between intraoral drainage and longer LOS should be interpreted cautiously. The route of drainage is selected according to anatomical accessibility, infection extent and clinical judgement; the observed association may therefore reflect confounding by indication rather than an adverse effect of the intraoral approach. Similarly, immediate extraction may be more feasible in less complex cases, whereas postponed extraction may reflect clinical instability or anatomical complexity. In a 248-patient cohort, Heim et al. found that removal of the odontogenic focus performed simultaneously with abscess incision was associated with the shortest LOS, while later extraction during the same admission was less favourable [32]. Differences in timing definitions and case selection, however, preclude a direct causal comparison with the present immediate and postponed extraction categories. Prompt drainage and elimination of the odontogenic focus nevertheless remain central management principles [3, 6, 28, 29, 32, 33]. From a systems perspective, the results support early multidisciplinary assessment involving oral and maxillofacial surgery, anaesthesia and intensive care when airway compromise or extensive spread is suspected, consistent with large inpatient and longitudinal series documenting the ICU burden of severe odontogenic infection [7, 8]. The multivariable findings additionally highlight recorded pre-existing disease and delayed presentation as potential factors relevant to early risk assessment. However, the present analysis was designed to identify independent associations rather than to develop a clinical prediction model. Development of a clinical risk score would require dedicated model development, internal validation and subsequent external validation before routine clinical use.

Socioeconomic status, dental access and treatment costs were not assessed in this study. These factors may be particularly relevant in view of the independent association observed between delayed presentation and prolonged hospitalisation. An Italian study linked severe odontogenic infections with financial hardship, while recent English data demonstrated an association between socioeconomic deprivation and admission for odontogenic cervicofacial infection [34, 35]. Future studies should therefore investigate whether barriers to timely dental assessment and referral contribute to delayed presentation and subsequent inpatient resource use. The present study should be viewed as contemporary clinical corroboration in a German, single-centre, odontogenic-only cohort rather than as evidence of a novel standalone prediction model. Its results remain relevant to public and professional awareness because earlier dental assessment, recognition of airway warning signs and timely referral may reduce the burden of severe infections [3, 29]. The strong independent association between delayed presentation and prolonged hospitalisation further supports investigation of timely access to dental and specialist care in future prospective studies. Several limitations must be acknowledged. The retrospective single-centre design entails risks of selection bias, incomplete documentation and limited generalisability. Comorbidity and symptom variables were chart-derived; the exact diagnostic composition of the broader recorded pre-existing disease field was not stratified, and uniform objective thresholds for dyspnoea, dysphagia and trismus were unavailable. Antibiotic agent and duration were not evaluated as study variables. Institutional anatomical terminology may limit comparability. Timing depended on day-level documentation, and the near-equivalence of symptom-to-presentation and symptom-to-operation intervals precludes a separate estimate of in-hospital delay. Although multivariable modelling was performed to account for several clinically relevant potential confounders, residual confounding cannot be excluded. The relatively modest sample size resulted in wide confidence intervals for several adjusted estimates, particularly for recorded pre-existing disease and anatomical variables, limiting the precision of these estimates. Furthermore, multiple unadjusted comparisons were performed and should therefore be interpreted cautiously and primarily as exploratory. Prospective multicentre validation is required before the identified associations can be used for individual clinical prediction or incorporated into a clinical risk score.

## Conclusion

Several demographic, clinical, anatomical and laboratory characteristics were associated with prolonged hospitalisation in unadjusted analyses. However, after multivariable adjustment, recorded pre-existing disease and longer time from symptom onset to hospital presentation remained independently associated with prolonged LOS. In particular, delayed presentation showed a strong association with extended inpatient care, whereas age, multi-space infection, submandibular and floor-of-mouth involvement, dyspnoea and CRP did not remain independently associated after adjustment. ICU admission, tracheostomy, drainage route and extraction timing should be interpreted primarily as treatment-course markers rather than independent admission predictors. These findings highlight the potential relevance of comorbidity and timely presentation for early risk assessment, while prospective multicentre studies are required to validate their prognostic utility.

## Data Availability

The datasets generated and analyzed during the current study are not publicly available due to institutional data protection policies and military confidentiality regulations but are available from the corresponding author upon reasonable request.
